# Effect of Dietary Supplementation with a Mixture of Natural Antioxidants on Milk Yield, Composition, Oxidation Stability and Udder Health in Dairy Ewes

**DOI:** 10.3390/antiox12081571

**Published:** 2023-08-06

**Authors:** Agori Karageorgou, Maria Tsafou, Michael Goliomytis, Ariadni Hager-Theodorides, Katerina Politi, Panagiotis Simitzis

**Affiliations:** Laboratory of Animal Breeding and Husbandry, Department of Animal Science, Agricultural University of Athens, Iera Odos 75, 11855 Athens, Greece; akarageorgou@aua.gr (A.K.); mgolio@aua.gr (M.G.); a.hager@aua.gr (A.H.-T.); katerinapoliti@aua.gr (K.P.)

**Keywords:** antioxidants, lymphocyte, macrophage, milk yield, plant bioactive compounds, polyphenols, oxidative stability, polymorphonuclear leukocytes, somatic cell count

## Abstract

Due to the limitations in the use of antibiotic agents, researchers are constantly seeking natural bioactive compounds that could benefit udder health status but also milk quality characteristics in dairy animals. The aim of the current study was therefore to examine the effects of a standardized mixture of plant bioactive components (MPBC) originated from thyme, anise and olive on milk yield, composition, oxidative stability and somatic cell count in dairy ewes. Thirty-six ewes approximately 75 days after parturition were randomly allocated into three experimental treatments, which were provided with three diets: control (C); without the addition of the mixture, B1; supplemented with MPBC at 0.05% and B2; supplemented with rumen protected MPBC at 0.025%. The duration of the experiment was 11 weeks, and milk production was weekly recorded, while individual milk samples for the determination of composition, oxidative stability, somatic cell count (SCC), pH and electric conductivity were collected. Every two weeks, macrophage, lymphocyte, and polymorphonuclear leukocyte counts were also determined in individual milk samples. It was observed that milk yield was the greatest in the B2 group, with significant differences within the seventh and ninth week (*p* < 0.05), whereas no significant differences were found for milk composition, with the exception of the seventh week, when protein, lactose and non-fat solid levels were lower in MPBC groups (*p* < 0.05). Oxidative stability was improved in the groups that received the MPBC, with significant differences at the third, seventh, tenth and eleventh week (*p* < 0.05). SCC was also significantly lower at the second, eighth and ninth week in B2 compared to the other groups (*p* < 0.05), while no significant effects on the macrophage, lymphocyte, and polymorphonuclear leukocyte counts were observed. In conclusion, the MPBC addition had a positive effect on sheep milk yield, oxidative stability and somatic cell count, without any negative effect on its composition.

## 1. Introduction

There is an increased public awareness of potential health hazards induced by the excessive use of in-feed antibiotics that is reflected in the legislation of many countries, such as the EU [1] and the efforts of animal scientists to find alternative safe natural feed additives [2,3]. Consumer concerns refer to toxicity, residues and metabolites in milk that can induce bacterial resistance in human infections. In general, farmers and industry comply with the legislation and several food safety controls are routinely carried out; however, these concerns remain possibly as a result of incorrect information [4,5]. Currently, research efforts are focused on the development of functional dairy products that fortify human health and are in harmony with the concept of sustainable production, green economy, environmental protection, and proper health and welfare status of dairy animals [2]. Bioactive compounds of plants, well known as phytobiotics, possess strong antioxidant and anti-inflammatory properties that depend upon their type and amount and are generally cheaper and safer compared to synthetic antibiotic agents [6]. Their dietary supplementation generally improves performance and ameliorates the health status of ruminants due to their multifaceted properties [6]. Milk Somatic Cell Count (SCC) is often used as an indirect index of mammary health, since high values are strongly related with changes in milk quality, poor udder health and inflammatory damage of mammary tissue leading to significant economic losses in modern dairy farms [7].

At the same time, the dietary inclusion of plants’ bioactive compounds improves milk oxidative stability, resulting in dairy products of high-quality and safety [8,9]. An antioxidant is defined as any substance that when present in low concentrations compared to the oxidizable substrate (i.e., proteins, lipids, carbohydrates and DNA) significantly delays or prevents oxidation of this substrate through its radical scavenging, metal ion chelation, and singlet oxygen quenching properties [10]. A compound exerts its antioxidant activities by inhibiting the creation of reactive oxygen species (ROS), or directly cleaning free radicals [10]. ROS are small molecules that contain active oxygen and are produced as by-products in sub-cellular organelles such as mitochondria. A high concentration of ROS in any normal cell can turn it into a malignant cell [10]. Phytobiotic supplementation may be more efficient in animals that are under physiologic stress, such as the peak of milk production in dairy animals [11]. In addition, the combination of phytobiotics from different plants may exert an increased antioxidant activity due to their synergism [12]. However, several parameters, namely area of origin, period of harvesting within the year, used part of the plant (leaf, bark, seeds or root) and method of isolation (steam distillation, extraction with non-aqueous solvents, cold expression, etc.) modify the antioxidant capacity and the efficacy of each phytogenic compound. Discrepancies are also observed due to the type of the phytobiotic, its level of dietary supplementation, the composition and the digestibility of the basal diet, the level of feed intake and hygiene and environmental conditions [2].

The objective of the present study was to determine if the supplementation with a mixture of plant bioactive components (MPBC) to high-producing dairy ewes that are prone to subclinical udder health disorders may alleviate this stressful condition and, consequently, improve their lactation performance and health status.

## 2. Materials and Methods

### 2.1. Animals

Thirty-six 2-year-old Chios ewes with similar body condition scores (2.5–3.0) and a mean weight of 52.3 ± 1.9 kg that were at their second parity and thirty days after lamb weaning (75 ± 5 days after lamb birth) were randomly selected from the sheep herd of the experimental farm of the Agricultural University of Athens and allocated into three experimental groups of twelve ewes each based on their milk yield and body weight. The ewes of the flock were mated following estrus synchronization with Ovigest intravaginal progestogen sponges ( Hipra S.A., Girona, Spain).

All animals initially consumed alfalfa hay and the same concentrate basal diet (Table 1) without the addition of MPBC for one week in order to become acclimatized to the experimental conditions. After this adaptive pre-experimental period, they received the three experimental diets for 11 weeks. One of the groups served as a control (C) and was fed with the previous concentrate diet, whereas the other two groups were offered the same concentrated diet further supplemented with MPBC (B1) at the level of 0.05% or with rumen protected MPBC at the level of 0.025% (B2). These levels were selected based on our preliminary studies. Furthermore, we attempted to evaluate whether a lower level of supplementation in a rumen protected form would return comparable results with the 0.05% level in the framework of precision livestock feeding. The MBPC used in this trial (NuPhoria, Nuevo S.A., Schimatari, Viotia, Greece) was a proprietary mixture of phytogenic substances originating from thyme (*Thymus vulgaris*), anise (*Pimpinella anisum*) and olive (*Olea europea*) at an approximate ratio of 20, 35 and 45% with a standardized active ingredient concentration of 100 g/kg. In detail, the levels of thymol, anethole and hydroxytyrosol in thyme, anise and olive were 24, 210 and 50 g/kg, respectively. Rumen-protected MPBC was obtained by freeze drying with maltodextrin at a rate of 50:50. Maltodextrins of different dextrose equivalents are commonly used as encapsulating agents due to their high-water solubility, low viscosity and colorless solutions [13,14]. Ewes in the present study consumed on average 2.0 kg of feed (concentrate and forage at a mean ratio of 50:50). Quantities of concentrates were constantly adjusted to the milk yield of each ewe and the additional demanded dry matter for high yielding animals was individually provided.

Ewes were housed in 3 different pens (one pen per treatment) at the premises of the Agricultural University of Athens. Each pen consisted of an indoor and outdoor area and had the same direction and orientation, the same covered area (3 m^2^/ewe) and was equipped with similar troughs for feeding (12 individual feeders indoors for concentrate and 1 feeder outdoors for alfalfa hay per pen). Water was available ad libitum and the diet that was formulated according to ewes’ individual requirements, based on their body weight and milk yield, was provided twice daily at 8 a.m. and 15 p.m. Forage was offered to the animals after assuring that the concentrate was completely consumed. No refusals of forage and/or concentrates were observed.

### 2.2. Determination of Milk Yield Composition and Oxidative Stability

Ewes were milked twice per day (6:00 a.m. and 18:00 p.m.) in a 12 stall milking parlor (GEA Westfalia, Düsseldorf, Germany). A pulsation ratio of 50:50 was applied; pulsation rate was 150 cycles min^−1^ with 37.5 kPa vacuum level. Milk yield, determined as the sum of the morning and afternoon milking, was recorded on day 1 prior to and on week 1–11 after MPBC dietary supplementation. Fat corrected (FCM6%) milk yield was also calculated using the following formula:

Fat corrected milk (FCM) in 6% (FCM6%) = (0.28 + 0.12 × milk fat concentration (%)) × milk yield (kg/d).

Individual milk samples were also collected on acclimation week and on week 1–11 after MPBC dietary supplementation and analyzed for fat, protein, lactose, total solids-not-fat, pH, electric conductivity and somatic cell count by using the Lactoscan COMBO Milk Cell Analyser (Lactoscan, Nova Zagora, Bulgaria) in accordance with international standard protocol guidelines. Milk oxidative stability was evaluated by measuring the levels of malondialdehyde (MDA), a secondary lipid oxidation product formed by hydrolysis of lipid hydroperoxides. MDA concentration (ng/mL) was determined by applying a selective third-order derivative spectrophotometric method, previously developed by Botsoglou et al. [15].

### 2.3. Isolation of Milk Somatic Cells and Milk Somatic Cell Immunophenotyping

Every two weeks, 15 mL individual milk samples were also collected and kept on ice for the determination of macrophage, lymphocyte, and polymorphonuclear leukocytes count. Milk somatic cells (MSC) were isolated following a modified protocol of Koess and Hamann [16] optimized for sheep milk. Briefly, milk samples were centrifuged at 400× *g* for 15 min at 4 °C. Pellets were resuspended in 15 mL of dilution buffer, i.e., phosphate-buffered saline (PBS; pH 7.4) containing 0.01% sodium azide (NaN3) and 0.2% bovine serum albumin (BSA). Samples were centrifuged at 400× *g* for 10 min at 4 °C. Pellets were resuspended in 4 mL of dilution buffer and centrifuged at 400× *g* for 10 min at 4 °C. Cell pellets were resuspended in 1 mL of dilution buffer and filtered through 40 μm cell strainers.

Cell surface labelling was performed with anti-CD11b, anti-CD8 and anti-Cytokeratins 4 + 5 + 6 + 8 + 10 + 13 + 18 (anti-Pan Cytokeratins) for the identification of granulocytes and macrophages, T-cytotoxic and epithelial cells, respectively. In addition, propidium iodide (PI) staining was used to differentiate live from dead cells. Aliquots of MSC prepared as described above containing approximately 2 × 10^5^ cells were centrifuged at 400× *g* for 5 min at 4 °C and cell pellets were resuspended in 50 μL ice cold antibody solutions containing combinations of 0.002 mg/mL anti-CD11b conjugated to Fluorescein isothiocyanate (FITC), 0.005 mg/mL anti-pan Cytokeratins conjugated to Allophycocyanin (APC) and 0.002 mg/mL CD8 R-PE antibodies. Cells in the staining solutions were incubated on ice and in the dark for 30 min, then a 2 mL dilution buffer was added, samples were centrifuged at 400× *g* for 5 min and cell pellets were resuspended in 100 μL dilution buffer. DNA staining was performed by addition of PI at a final concentration of 5 ng/μL. Following 10 min incubation at room temperature in the dark, 100 μL of PBS were added and samples were analyzed by flow cytometry (Cytomics FC 500, California, Beckman Coulter Inc., Fullerton, CA, USA).

Instrument voltage/gain for detectors FS, SS, FL1, FL2, FL3, FL4 and FL5 were set at 700/2.0, 680/20.0, 550/1.0, 650/1.0, 650/1.0, 614/1.0, and 601/1.0, respectively. The samples were run at medium speed and approximately 65,000 events were collected per sample. Data were stored as list mode files. Events that were identified as being of appropriate size and granularity based on their position on an FS/SS dotplot and were negative for PI were considered as live cells. PI stains nucleic acids only in cells with disrupted cell membranes, i.e., necrotic cells and thus cells that were negative for PI staining were considered as live cells. Dead cells were not included in further analysis as they often exhibit non-specific staining with cell surface antibodies and may be misclassified. Live cells that stained positive for CD11b (FL1) with higher SS values were classified as polymorphonuclear granulocytes (PMN) and live cells that stained positive for CD11b with lower SS values were classified as monocytes/macrophages (MPh). Epithelial cells were identified from the CD11b−/CD8− live cells that stained positive for pan-cytokeratins. Lymphocytes were identified as the live cells that were positioned in the FS/SS dotplot in the area identified by CD8+ cells. Proportions of each cell subset in the MSC were estimated as a percentage of the live cells.

### 2.4. Statistical Analysis

The experimental unit was the animal since it was the smallest unit upon which either the treatment was applied or the measurements were made. Data were subjected to repeated measures analysis of variance using the MIXED procedure of SAS software, with dietary treatment as the fixed factor and sampling week as the repeated factor. SCC was log transformed prior to statistical analysis in order to achieve normal distribution. Significant differences were tested with Bonferroni adjustment at 0.05 significance level and the results are presented as least square means ± S.E.M.

## 3. Results

**Figure 1:**
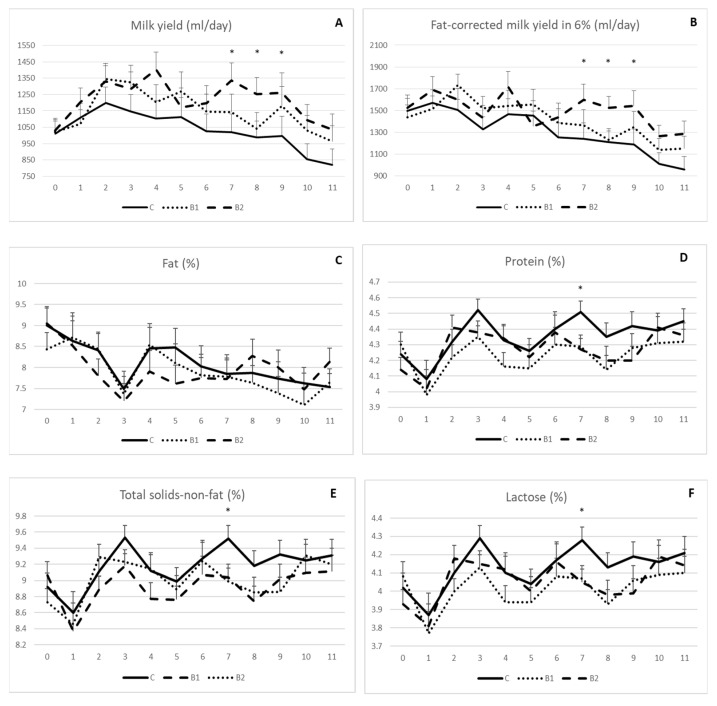
Effect of MPBC dietary supplementation on milk yield (**A**), fat-corrected milk yield in 6% (**B**), fat (**C**), protein (**D**), total solids-not-fat (**E**) and lactose (**F**). Control group was fed with the basal concentrate diet (C), whereas the other two groups were offered the same concentrated diet further with MPBC (B1) at the levels of 0.05% or with rumen protected MPBC (B2) at the levels of 0.025%. The use of (*) indicates significant difference at *p* < 0.05.

As shown, the milk yield (Figure 1A) and fat-corrected milk yield in 6% (Figure 1B) were generally higher in the MPBC supplemented groups than the controls with significant differences between the 7th and 9th week of the experiment for the B2 compared to the other groups (*p* < 0.05). In detail, the milk yield was 1335, 1021 and 1142 (±109) mL/day on week 7, 1253, 988 and 1042 (±99) mL/day on week 8 and 1262, 996 and 1179 (±121) mL/day on week 9 for the B2, C and B1 groups, respectively (*p* < 0.05; Figure 1A). The respective values for the fat-corrected milk yield were 1599, 1243 and 1367 (±142) mL/day on week 7, 1525, 1209 and 1230 (±104) mL/day on week 8 and 1543, 1187 and 1348 (±14,121) mL/day on week 9 for the B2, C and B1 groups, respectively (*p* < 0.05; Figure 1B).

Milk composition was generally not affected by MPBC dietary supplementation. No significant differences were shown for milk fat throughout the experiment (Figure 1C). Similar findings were observed for milk protein (Figure 1D), total solids-non-fat (Figure 1E) and lactose (Figure 1F) with the only exception at the 7th week, when the values for these parameters were higher in the control compared with the MPBC supplemented groups (*p* < 0.05). In detail, milk protein (%) was 4.51, 4.29 and 4.27 (±0.07), milk total solids-non-fat (%) were 9.52, 9.04 and 8.99 (±0.16) and milk lactose was 4.28, 4.07 and 4.05 (±0.07) for the C, B1 and B2 groups, respectively.

**Figure 2:**
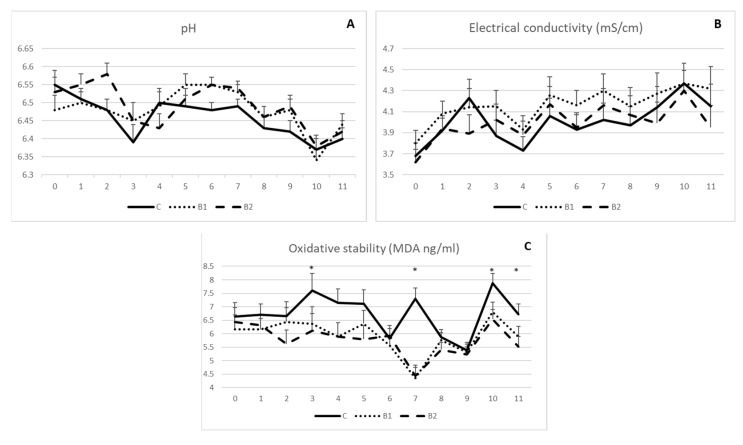
Effect of MPBC dietary supplementation on milk pH (**A**), electrical conductivity (**B**) and oxidative stability (MDA levels) (**C**). Control group was fed with the basal concentrate diet (C), whereas the other two groups were offered the same concentrated diet further supplemented with MPBC (B1) at the levels of 0.05% or with rumen protected MPBC (B2) at the levels of 0.025%. The use of (*) indicates significant difference at *p* < 0.05.

As indicated in Figure 2A,B, milk pH and electrical conductivity were not influenced by MPBC dietary supplementation, since no significant differences were observed among the experimental groups during the 11-week experimental period. On the other hand, milk oxidative stability was in general improved in MPBC supplemented groups as shown by the reduced MDA values. Significant differences were shown on week 3, 7, 10 and 11 (*p* < 0.05; Figure 2C). The respective MDA values (ng/g) were 6.36 and 6.11 vs. 7.60 (±0.64) on week 3, 4.35 and 4.44 vs. 7.29 (±0.40) on week 7, 6.81 and 6.54 vs. 7.87 (±0.36) on week 10 and 5.89 and 5.52 vs. 6.72 (±0.29) on week 11 for the B1, B2 and C groups, respectively (*p* < 0.05; Figure 2C).

**Figure 3:**
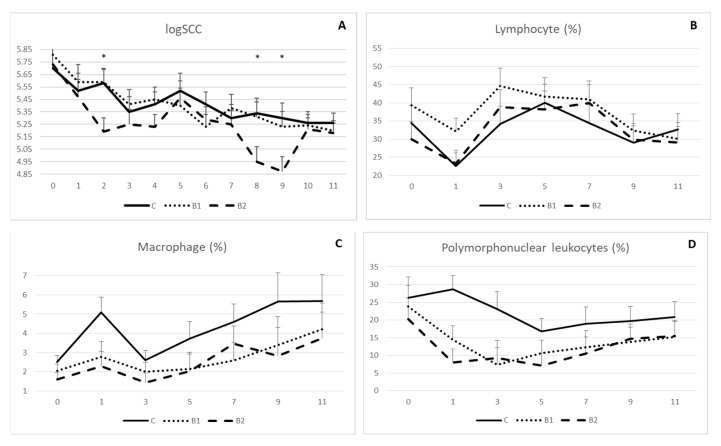
Effect of MPBC dietary supplementation on logSCC (**A**), lymphocyte (**B**), macrophage (**C**) and polymorphonuclear leukocytes (**D**) count of sheep milk. Control group was fed with the basal concentrate diet (C), whereas the other two groups were offered the same concentrated diet further with MPBC (B1) at the levels of 0.05% or with rumen protected MPBC (B2) at the levels of 0.025%. The use of (*) indicates significant difference at *p* < 0.05.

Rumen protected MPBC dietary supplementation decreased milk somatic cell count, as indicated in Figure 3A. However, significant differences were shown on week 2, 8 and 9. In detail, logSCC was 5.19 vs. 5.58 and 5.59 (±0.11) on week 2, 4.95 vs. 5.34 and 5.31 (±0.12) on week 8 and 4.87 vs. 5.30 and 5.23 (±0.12) on week 9 for B2, C and B1 group, respectively (*p* < 0.05; Figure 3A). On the other hand, proportions for lymphocyte (Figure 3B), macrophage (Figure 3C) and polymorphonuclear leucocytes (Figure 3D) were not significantly different among the experimental groups, although values for macrophage and polymorphonuclear leucocytes were numerically higher in controls compared to the MPBC dietary supplemented groups throughout the experiment.

## 4. Discussion

As indicated by the findings of the present study, the milk yield and fat-corrected milk yield in 6% were generally higher in the MPBC supplemented groups than the controls with significant differences between the 7th and 9th week of the experiment for the B2 compared to the other groups. However, milk composition was not significantly affected by MPBC dietary supplementation, while oxidative stability was improved in B1 and B2 groups. According to the existing literature, thyme and/or celery seed mixture [17], thyme or celery essential oil [18], cornus extract enriched with EOs of oregano and thyme [19], orange peel essential oil (EO) [20] and EO components mixture (thymol, eugenol, vanillin, guaiacol and limonene) [21] induced an increase in the milk yield in dairy ewes. Moreover, Kholif et al. [22] reported that capsicum/thymus essential oils blend at 2 mL and/or enzymes cocktail at 4 g per day enhanced milk yield and milk fat levels in dairy ewes. On the other hand, no effect of citral oil [23] or anise, clove, and thyme EO [24] on milk yield was observed, while an increase in milk yield, protein and fat levels was observed in dairy goats as a result of Boswellia sacra resin [25] and rosemary or lemon grass [26] dietary supplementation. Feed efficiency, milk yield and levels of protein, fat, total solids were increased as a result of Lippia alba hay inclusion in the diet of dairy goats [27]. Choubey et al. [28] observed that the dietary supplementation with flowers, shoots and leaves of *Woodfordia fruticosa*, the whole plant of *Solanum nigrum* and the seeds of *Trigonella foenum-graecum* improves antioxidant status in adult goats. In contrast, Leparmarai et al. [29] showed that grape seed dietary supplementation did not influence milk yield, milk composition and blood antioxidant status in dairy sheep and goats.

Shabtay et al. [30] reported that the addition of pomegranate extract to dairy cow diets resulted in higher milk production. Enhanced daily outputs of milk, energy corrected milk and fat were also observed after coriander oil dietary supplementation without any negative effect on cow health [31]. Greater milk yield, total solids, protein, lactose and fat and decreased malondialdehyde values were observed in dairy cows supplemented with a phytogenic feed additives mixture that contained menthol, anethole and other terpinenes [32]. Moreover, according to a meta-analysis by Belanche et al. [33], long-term exposure to a commercial blend of EOs (Agolin) resulted in a slight increase in milk yield at the level of 4%, while no effects on feed intake and milk composition were evident. Braun et al. [34] suggested that the aforementioned effects on milk production in dairy cattle could be attributed to the improved rumen fermentation, feed efficiency, nutrients’ absorption and utilization, and increased uptake of cations like calcium and ammonium as a result of phytobiotics’ dietary supplementation. As indicated, herbs and their extracts can accelerate digestion by reducing residence time in the digestive tract [35], while the observation of similar values in feed intake among treatments may indicate that although the MPBC dietary supplementation improved nutrient digestibility, did not negatively affect feed palatability and acceptance. However, no effects of thyme oil and thymol [36], eucalyptus, thyme and anise oil [37], eugenol [38], cinnamaldehyde and eugenol [39], cinnamaldehyde and garlic oil [40], garlic or juniper berry EO [41], oregano leaves [42,43], EOs components mixture (thymol, eugenol, vanillin, guaiacol and limonene) [44,45,46,47], blend of oregano, cinnamon, thyme and orange peel EOs [48] and mixture of eugenol, geranyl acetate and coriander oil [49] on milk yield and composition of dairy cattle are observed. In a study carried out with a mixture of plant bioactive components, a decrease in milk fat content was observed, while the other milk components were not affected in dairy cows [50]. In water buffaloes, dietary supplementation with a phytogenic mix containing seeds of fennel, ajwain and fenugreek, tubers of ginger, leaves of *Swertia chirata*, roots of licorice, fruits of *Citrullus colocynthis*, *Terminalia chebula* and turmeric did not affect milk yield and rumen fermentation parameters, apart from pH [51].

Moreover, the improved milk oxidative stability as suggested by the reduced MDA levels indicating that the provision of polyphenols with potent antioxidant or co-antioxidant activity might be a beneficial strategy to protect mammary cells against the adverse effects of free radicals. Part of the molecular multifunctionality of the natural bioactive compounds is their antioxidant capacity, which improves the immune status and reduces the oxidative stress of animals [52,53,54]. Phenolic compounds have been found to in vitro modify immune status via the downregulation of the inflammatory response, since they reduce the production of cytokines and reactive oxygen species, as well as the functionality of cytotoxic T-lymphocytes and natural killer cells [55]. The anti-inflammatory and antioxidant activity of polyphenols has been confirmed by in vivo studies in dairy cows, goats and ewes [52,56,57]. The aforementioned properties are attributed to their ability to chelate with free radicals, inhibit the enzymes actions associated with the mechanisms of oxidative stress, reinforce the functionality of antioxidant mechanisms, and prevent the lipid oxidation [53,58,59,60].

As indicated, SCC was reduced in dairy ewes that were dietary supplemented with the rumen protected MPBC with significant differences on week 2, 8 and 9. The decreased levels of SCC in milk from ewes dietary supplemented with MPBC is associated with an ameliorated udder health status, since milk SCC is considered as an index of mammary health. This finding could be attributed to the provision of several hydrophylic and lipophylic phenols that are included in the MBPC and through their antioxidant properties fortify mammary cells against the adverse effects of free radicals produced as a result of oxidative stress. Similar findings were reported by Hashemzadeh-Cigari et al. [50] in dairy cows after their supplementation with a phytobiotics-rich herbal mixture (185 g/cow) that contained cinnamon bark, turmeric roots, rosemary leaves and clove buds. Moreover, supplementation of rosemary extract [59] or a mixture of essential oils [21] to lactating ewes and concentrated pomegranate extract to dairy cows [30] resulted in reduced milk SCC. Jaquezeski et al. [60] also found that curcumin dietary supplementation improved milk yield and antioxidant capacity, while a reduction in somatic cell count and protein oxidation was reported in dairy sheep. In dairy cows, thyme essential oil supplementation via esophageal tube decreased the standard plate count, while no differences were observed in the raw milk composition [61]. Moreover, although Rodrigues et al. [62] observed an increase in milk yield after dietary supplementation with a phytogenic mix, no effect on milk composition and incidence of clinical mastitis was observed in dairy cows.

Although SCC was decreased as an effect of rumen-protected MPBC dietary supplementation, no significant differences were observed among the treatments concerning the macrophage, lymphocyte, and polymorphonuclear leukocyte (PMN) counts. However, although there was not a clear tendency for the lymphocyte count, macrophages were increased and polymorphonuclear leukocytes were decreased from the beginning till the end of the experimental period. Although only numerical, MPBC supplemented groups had a lower PMN count compared to the controls, indicating a tendency for a healthier mammary gland, since PMN being the principal leucocytes that are increased during pathogen invasion, are closely correlated with high SCC [63] and oxidation stress in mammary gland [64]. On the other hand, macrophages represents 5–7% of leucocytes in ewe milk and are found to minimally contribute to the proteolytic activity in ewe milk [65]. Finally, lymphocytes represents approximately 40% of leucocyte population [66] and an absence of differences in the lymphocyte count as an effect of SCC suggests that in ewe milk this population is quite stable [63].

## 5. Conclusions

Plant extracts have been widely recognized as potential functional alternatives to antibiotics due to their green, safe, and efficient properties. Dietary supplementation with a mixture of phytogenic substances originated from thyme, anise and olive was effective in improving milk oxidative stability (third, seventh, tenth and eleventh week), enhancing performance (seventh–ninth week) and lowering SCC (second, eighth and ninth week) when provided in its rumen protected form in mid-lactation high-producing dairy ewes. Considering the positive outcome on milk yield, oxidative stability and SCC and the lack of any side effects on the other milk properties, the MPBC used in the present study and especially in its rumen protected form appeared as a promising candidate for a feed additive for dairy ewes. The use of the protected form of MPBC may be successfully adapted in a production system that incorporates precision livestock feeding. However, a further in-depth analysis is necessary regarding its production cost before establishing its regular use.

## Figures and Tables

**Table 1:** Composition and analysis of dairy ewes’ diet.

Components (g/kg)		
Corn	234	
Wheat	175	
Barley	175	
Soybean Meal (44%)	182.5	
Sunflower Meal (28%)	50	
Wheat Bran	150	
Sodium Chloride (NaCl)	10	
Limestone	18.5	
Monocalcium Phosphate	4	
Vitamins & Trace elements Premix *	1	
Calculated Analysis	Concentrates	Alfalfa hay
Dry Matter—DM (%)	86.0	93.5
Crude protein—CP (%)	17.0	10.2
Crude Fiber (%)	6.0	34.2
Ash (%)	6.5	7.4
Fat (%)	2.1	2.3
Calcium (%)	0.9	-
Phosphorus (%)	0.6	-
Sodium (%)	0.4	-

* Premix contained per kg: 25 g Mn, 30 g Fe, 45 g Zn, 0.10 g Se, 0.50 g Co, 1.75 g I, 10,000 kIU vitamin A, 2000 kIU vitamin D3, 20 kIU vitamin E (kIU: 1000 International Units).

## Data Availability

All data used to support the findings of this study are included within the article.
